# WHO water quality standards Vs Synergic effect(s) of fluoride, heavy metals and hardness in drinking water on kidney tissues

**DOI:** 10.1038/srep42516

**Published:** 2017-02-14

**Authors:** Hewa M. S. Wasana, Gamage D. R. K. Perera, Panduka De S. Gunawardena, Palika S. Fernando, Jayasundera Bandara

**Affiliations:** 1 National Institute of Fundamental Studies, Hantana Road, CP 20000, Kandy, Sri Lanka; 2 Ministry of Social empowerment and Welfare,Department of Divineguma Development, Peradeniya, Sri Lanka; 3 Department of Veterinary Pathobiology, Faculty of Veterinary Medicine and Animal Science, University of Peradeniya, Peradeniya, Sri Lanka; 4 Division of Pathobiology, Veterinary Research Institute, Gannoruwa, Sri Lanka

## Abstract

Despite WHO standards, waterborne diseases among the human being are rising alarmingly. It is known that the prolong exposure to contaminated water has major impact on public health. The effect of chemical contaminations in drinking water on human being is found to be chronic rather than acute and hence can be defined “consumption of contaminated drinking water could be a silent killer”. As the WHO recommended water quality standards are only for individual element and synergic effects of trace metals and anions have not been considered, investigation of synergic effects of trace metals and anions and their effect on human being is of prime important research. By an animal trial, we investigated the synergic effect(s) of heavy metals, aluminium, arsenic, fluoride and hardness in drinking water on kidney tissues of mice. Our investigation strongly suggests existing of a synergic effect especially among Cd, F and hardness of water which could lead to severe kidney damage in mice, even at WHO maximum recommended levels. Hence, the synergic effect(s) of trace metals, fluoride and hardness present in drinking water should be investigated meticulously when stipulating the water quality at WHO maximum recommended levels.

Contamination of freshwater resources by naturally occurring phenomena or chemical wastes due to rapid industrial growth and urbanization is one of the major ecological concerns of the contemporary world. The biggest water pollutants are heavy metals, nitrate, arsenic, fluoride and synthetic chemical emissions especially due to industrialization^1^,^2^,^3^. Quality of drinking water has a major influence on public health and prolong exposure to contaminated water has been known to increase the risks of cancer and disorders in kidney, liver and reproductive organs, etc^1^,^4^,^5^. The effect of chemical contaminations in drinking water on human being is found to be chronic rather than acute. Therefore, understanding of water quality on public health is vital because waterborne diseases are still a major cause of death in many parts of the world^6^,^7^. Though the quality of drinking water is regulated by the WHO stipulated standards, water-related disease is complex and diverse in nature. The recommended WHO water quality standards are only a guideline and cannot be considered as the safe level of contaminants especially for the tropical countries due to consumption of excess water due to hot-dry conditions. On the other hand, the WHO recommended water quality standards are only for individual element and chronic synergic effects of trace metals and anions have not been considered. Though, the acute synergic effects of trace metals have been reported, those studies do not mimic the natural environmental conditions. i.e. when Sprague–Dawley male rats were given cadmium (50 mg/L) and fluoride (100 mg/L) alone or in combination via drinking water for twelve weeks, an increased liver and kidney damages has been noted in cadmium and fluoride co-exposed rats compared with that exposed to cadmium or fluoride alone^8^.

As such, synergic effects of trace heavy metals and common anions present in water could be a major critical factor on public health and the investigation of interaction among trace metals and anions and their effect(s) on human being is of prime important research. Our earlier finding revealed a clear correlation between incidence of chronic kidney disease of unknown etiology (CKDu) and the water quality^9^. Thus, encourages us to investigate how the common trace metals and anions present in drinking water and their synergic effect(s) interfere on kidney tissues via animal model. In this investigation, we demonstrated how the trace metals together with fluoride and hardness present in drinking water at WHO maximum recommended levels may cause kidney damage by their synergic effect(s).

## Results and Discussion

### Behavioral and visible clinical changes of mice after treatment

The number of live-death counts of animals, body weight change and average water intake during test period are given in Table S3. In brief, there were no remarkable deviations of amount of water consumption among the test and the control groups. When considering weight changes, only mice in the control group had acquired a weight gain and the rest of mice in all other groups had weight loss in which mice in group 3 were the most affected. Except in control group, mice in other groups had dull coat and emaciation in several degrees. Moreover, in group 8, seven animals had severe itching with alopecia due to As toxicity^10^. Also in group 11, nine mice had severe itching with alopecia (Table S4). No cannibalism was noted among the mice in all groups. Deaths were observed in all groups except control group and group 10.

### Gross lesions

Presence of multifocal necrotic lesions on the kidneys and liver lobes were noted. Additionally, mild to moderate hepatomegaly and splenomegaly were observed in the affected mice, especially in groups 2, 3, 5, 7, 8, 9 and 11. Details are given in Table S4, Figures S2 and S3.

### Histopathological evaluations

**Figure 1:**
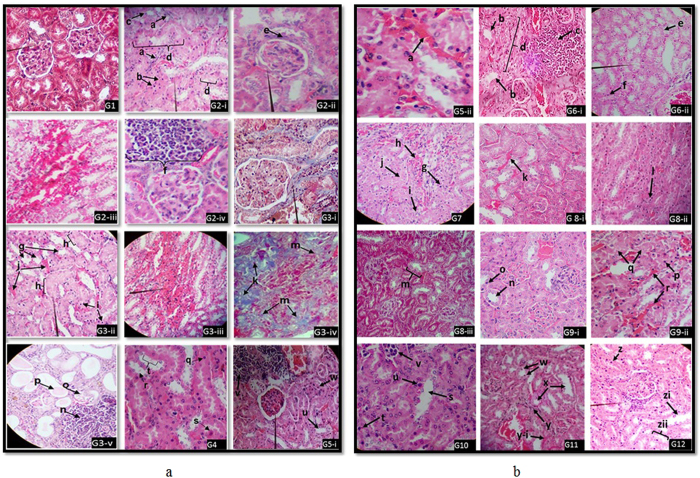
Histopathological evaluation of renal tissues in; **(a) G1-**Intact tubules, **pointer**-glomeruli and interstitium (H&E × 100). **G2-i** Tubular interstitial lesions, **a**-Formation of fibrous bands, **b**-Apoptotic cells with nuclear pyknosis, **c**- Tubular atrophy in proximal convoluted tubule (PCT), **d, Pointer** – Degeneration and necrosis of PCT (H&E × 100). **G2-ii-**Initiation of glomerular sclerosis, **e**-Fibrosis of Bow man capsules. **Pointer**–thicken Bow man capsule (H&E × 100). **G2-iii**-Hemorrhages at cortico-medullary area (H&E × 100). **G2-iv-**Interstitial inflammatory cell infiltration at renal cortex, **f**-infiltrated mononuclear inflammatory cells (H&E × 100). **G3-i** -interstitial fibrosis (blue colour, pointer) (MT × 100). **G3-ii**Tubular **lesion**s, **g-**Tubular atrophy (loss of brush borders with clear lumen in PTC, **h**-Degenerated and necrotic areas of PCT, **i**-Apoptosis of tubular epithelial cells with pyknotic nuclei, **j**-Hyperemic renal capillaries (H&E × 100). **G3-iii**-**pointer**-hemorrhagic area (H&E × 40). **G3-iv**-**k**-Global glomerular sclerosis (Blue colour), **m**-interstitial fibrosis (blue colour) (MT × 100). **G3-v-**Reduced functional renal tissue density and interstitial fibrosis, **n**-Mononuclear inflammatory cell infiltration, **o**-Tubular atrophy in PCT, **p**-Protein cast in a tubular lumen (H&E × 100). **G4-**Tubular lesions, **q**-Apoptotic cells with pyknotic nuclei PCT, **r**-Tubular atrophy (brush borders are lost with clear lumen) in PCT, **s**-degenerated tubular epithelial cell in a PCT, **t**-Necrotic area of a PCT (H&E × 100). **G5-i,u**-Tubular atrophy. **v**-Cell infiltration, **w**–Apoptotic cell with nuclear pyknosis, **pointer**-intact glomerulus (H&E × 40). **(b) G5-ii**, **a**-Hyperemic capillaries (H&E × 100). **G6-i**, **b**-Tubular atrophy in PCT (loss of brush borders with clear lumen), **c**-Mononuclear inflammatory cell infiltration, **d**-replacement of renal tissue with fibrous tissue in renal cortex (H&E × 100). **G6-ii, pointer–**PCT undergoing atrophy (loss of brush borders with clearing of the lumen) **e-**Degeneration and necrosis of tubules, **f**-Apoptotic cell with nuclear pyknosis (H&E × 40).**G7, g**-Apoptotic tubular epithelial cell, **h**-Hyperemic capillaries at cortical area, **i**-Initial stages of fibrosis, **j**-Degeneration and necrosis of PCT (H&E × 40). **G8-i, k-**Hyperemic capillaries (H&E × 100).**G8-ii,l-**Apoptotic cells of a PCT. **G8-iii, m-**Tubular degeneration and necrosis (MT × 40). **G9-i, n-**Tubular atrophy of a PCT, **o**-Apoptotic cells with nuclear pyknosis, (H&E × 100). **G9-ii, p**- Apoptotic cell. **Q-**Degeneration and necrosis of PCT, **r**-Hyperemic capillaries. (H&E × 100). **G10, s**-Tubular atrophy, **t**-Apoptosis, **u-**Degenerated cell of a PCT**, v-**Mononuclear cell infiltration (H&E × 100). **G11, w-**Apoptotic tubular epithelial cell, **x**-Tubular atrophy of PTC, y**-**Interstitial fibrosis, **y-i-**Hyperemic capillaries (H&E × 40).**G12, z**- Apoptotic cell, **zi**-Tubular atrophy of a PCT, **Pointer**-Mononuclear cell infiltration, **zii-**Cell degeneration and necrosis (H&E × 100).

Renal biopsy is considered the gold standard to establish diagnosis, especially when the cause is not obvious from other investigations^11^. Hence, histopathological studies on the kidney tissues of treated mice and the control group were carried out to assess the changes/damages to the kidney tissues caused by test cations, anions, hard water and their synergic/antagonistic effects. Light microscopic images of kidney tissue sections stained with Haematoxylin (H) & Eosin (E) and Masson’s trichrome of test groups and the control groups are shown in Fig. 1a,b. A working classification based on Banff 97 Classification of Renal Allograft Pathology^12^ and other lesions related to kidney damage (criteria detailed in SI) was employed to study the kidney histopathology. By using the working classification as given in supporting information, the test groups and the control group were assessed histopathologically and summarized in Table 1, Tables S5 and S6 in SI. In assessing histopathologial results, only the most prominent lesions associated with CKD (i.e. Interstitial fibrosis, Tubular atrophy, Mononuclear cell interstitial infiltration) and most severe lesions associated with CKD (i.e. Focal segmental glomerular sclerosis, Global glomerular sclerosis) were considered and listed in Table 1. However, other lesions related to CKD such as Apoptosis, Cell degeneration, Cell necrosis, Hyperemia and Hemorrhages were listed in Table S5.

When mice were treated with drinking water containing typical Cd, Pb, As, Al, F and hardness levels well below the WHO standards (group 1-control group, Fig. 1a: G1), kidney tissues of the mice were free of gross lesions or any other lesions related to CKD. Furthermore, since no any other symptoms or kidney damage was observed in the control group with the given diet, it can be confirmed that the diet has no impact on the study. However, when the mice were treated with the hard drinking water containing F and Cd with the WHO maximum standard (group 2), appearance of common lesions associated with CKD such as interstitial fibrosis, mononuclear cell interstitial infiltration and tubular atrophy were noted. Few focal segmental glomerular sclerosis in kidney tissues were also noted as shown in Fig. 1a, G2-i, G2-ii and G2-iii.The observation of initiation of focal segmental glomerular sclerosis (Fig. 1a G2-ii), along with the signs of tubular interstitial nephritis in kidney tissues strongly suggests the toxic severity of F, Cd and hardness combination, even when present in WHO maximum standards. To further substantiate the toxic synergic effect of F, Cd and hardness, animals were treated with the double dose of F, Cd and hardness combination, compared to WHO recommended levels. (group 3, Fig. 1a; G3-i to G3-v). More severe kidney damages were noted with much prominent lesions of the mice in the group 3, especially with marked global glomerular sclerosis and focal segmental glomerular sclerosis in the kidney tissues compared to mice in group 2. (Where mice were treated with WHO recommended levels of F, Cd and hardness.)

Nevertheless, when the mice were treated with drinking water, excluding one or two of the elements (i.e. either F, Cd or hardness), the focal segmental and global glomerular sclerosis lesions were absent. i.e. in groups 4, 5, 6 and 7, when mice were treated with drinking water containing; ***(a)*** Cd(0.003 mg/l) and hard water (385 mg/l) - group 4, Fig. 1a G4; ***(b**)* Cd (0.003 mg/l) and F (1.5 mg/l) - group 5, Fig. 1a,b; G5-i, G5-ii; ***(c)***F(1.5 mg/l) and hard water (385 mg/l)**-**group 6, Fig. 1b; G6-i and G6-ii) or ***(d)*** Cd (0.006 mg/l) alone - group 7, Fig. 1b; G7), focal segmental and global glomerular sclerosis were not observed in the kidney tissues of mice. However, interstitial fibrosis, mononuclear cell interstitial infiltration and tubular atrophy, still could be observed in kidney tissues of mice in groups 4, 5, 6 and 7 (Fig. 1a,b; G4, G5-i, G5-ii, G6-i, G6-ii and G7, although the damages are less severe compared to group 2. These results indicative of that the drinking water containing combination of Cd (0.003 mg/l)/F, (1.5 mg/l), Cd (0.003 mg/l)/hard water (385 mg/l), F(1.5 mg/l)/hard water (385 mg/l) and Cd (0.006 mg/l) alone may also lead to deterioration of the kidney tissues with the slow progression of the damage. The observation that, no lesions in kidney tissues of mice when mice were treated with F (0.05–10.00 mg/l) containing drinking water, indicate that the F (0.05–10.00 mg/l)) alone in drinking water does not cause CKD^13^.

Additionally mice were treated with drinking water containing; ***(e)***As, F, hardwater (0.015, 3.0, 385.0 mg/L, group 8, Fig. 1b; G8-i, G8-ii and G8-iii), ***(f)***Pb, F, hard-water, (0.015, 3.0, 385.0 mg/L, group 9, Fig. 1b; G9-i,G9-ii), ***(g)***Al, F, hard-water (0.200, 3.0, 385.0 mg/L, group 10, Fig. 1b; G10), ***(h)***As (0.015 mg/L. group 11, Fig. 1b; G11) and ***(i)*** drinking water containing WHO maximum standard levels of Al = 0.200, Cd = 0.003, As = 0.010, Pb = 0.010, F = 1.5 (mg/L) with relatively high hardness (CaCO_3_ = 200.0, MgCO_3_ = 185.0 mg/L, group 12, Fig. 1b; G12). Among these test groups, trivial kidney tissue damages were noticed in the cases of Al, F, hard-water treated group (group 10), while moderate kidney tissue damages were noticed in the cases of Pb, F, hard-water treated group (group 9). A substantial kidney damage was noticed with higher As dose (0.015 mg/L, group 11) treated mice and comparatively less kidney damage was noticed when mice were treated with As, F and hardness (group 8) compared to As only. Also, marked skin lesions(common in As treated mice)were noted both in As alone and As, F and hard-water treated mice (Figure S1-A,B,C). These results indicate that though the high doses of As is nephrotoxic, it is less nephrotoxic when present together with hardness and F. Interestingly, when mice were treated with drinking water containing WHO maximum standard levels of Al = 0.200, Cd = 0.003, As = 0.010, Pb = 0.010, F = 1.5 (mg/L) with relatively high hardness (CaCO_3_ = 200.0, MgCO_3_ = 185.0 mg/L, group 12, Fig. 1b; G12), an average interstitial fibrosis, mononuclear cell interstitial infiltration, tubular atrophy were noticed in kidney tissues of the mice and the kidney damage is less significant than that of drinking water containing WHO doses (group 2) or high doses (group 3) of Cd, F, hardness. Moreover, we have observed reduced nephrotoxic effect among mice in group 12 and thus, we believe a kind of antagonistic effect may responsible for this observation which needs further investigation.

**Figure 2:**
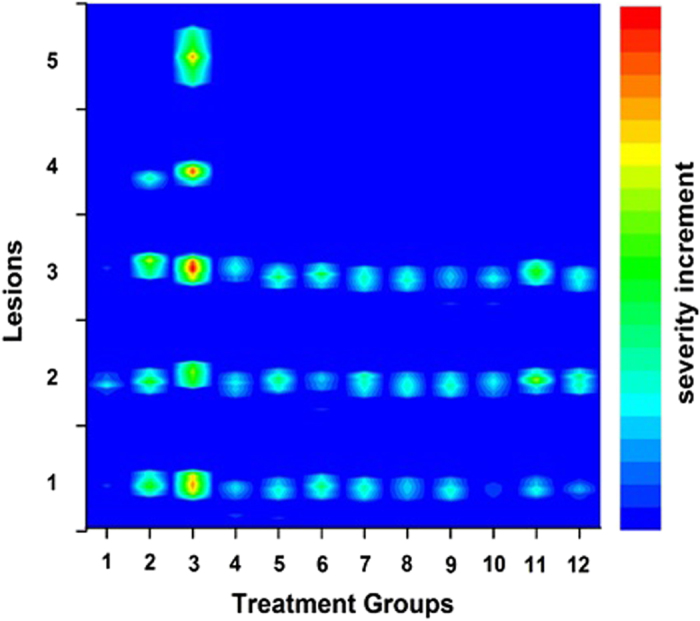
Severity increment of the CKD lesions of mice in test groups (2–12) and control group (1). (Lesions;1. Interstitial fibrosis 2. Mono-nuclear cell interstitial infiltration 3.Tubular atrophy, 4. Focal glomerular sclerosis and 5. Global glomerular sclerosis).

The pronounced toxic synergic effect(s) of heavy metals, hardness and F on CKD can be easily visualized by the 2-D plot of the observed histopathological changes in kidney tissues of mice against the treated conditions as shown in Fig. 2 (the details of how the Fig. 2 was plotted is given in SI). Only most severe and common CKD lesions were considered in plotting Fig. 2. The color code of the right hand side of Fig. 2 indicates the percentage of severity of the lesions. As shown in Fig. 2, control group is the least affected group and kidney tissues of mice in groups 2 and 3 are the mostly affected group where mice were treated with Cd, F and hard water. Additionally, kidneys of mice in groups 4, 5, 6 and 7 (excluding one or two of the elements i.e. either F, Cd or hardness), are less affected compared to groups 2 and 3. Also, the 2-D plot clearly demonstrates the non-synergic (antagonistic) effect of As, F, hardness (G8) compared to the mice in G11 where mice were treated only with As. Similarly, the synergic effect among G10 (Al, F, hardness) could not be observed compared to G6 (F and hardness).

The results presented above clearly indicate the toxicity of trace heavy metals, As, Al, F and hardness of drinking water on kidney tissues. Severity of the toxicity on kidney was found to depend on the contamination levels as well as their combined effects. Our investigation strongly suggests existing of a synergic effect(s) especially among Cd, F and hardness of water which could lead to severe kidney damage. The histopathological results clearly demonstrated that the mice fed with drinking water containing WHO recommended levels of Cd, F along with hardness, leading to early lesions similar to those of CKD. Toxic synergic effect of Cd, F and hardness is more pronounced when they are present in water at relatively higher concentrations (double concentrated as WHO maximum level). The toxicity effect of Cd-F, Cd-hardness, F-hardness or Cd alone on kidney is less pronounced than that of Cd,-F-hardness combination suggesting a synergic effect among Cd,-F-hardness. On the other hand, As alone has pronounced toxic effect on kidney than As-F-hardness at higher WHO standards, yet the toxicity of As or As-F-hardness on kidney is less compared to Cd-F-hardness. Apart from toxicity effect of As or As-F-hardness on kidney, skin lesions is also a common factor among in As or As-F-hardness treated mice. In conclusion, the severity of the toxicity of Cd, Pb, As, Al, F and hardness of drinking water, on kidney was found to depend on the contamination levels as well as their combined effects even at relatively low concentrations. Thus, these synergic effects even at WHO recommended maximum levels could be responsible for CKD, which needs further investigation when recommending WHO water quality.

Authors would like to make a special note here regarding CKDu prevailing in some part of the world and in Sri Lanka. As mentioned previously, several risk factors/ hypotheses have been proposed for the prevalence of CKDu in Sri Lanka^14^,^15^,^16^,^17^. Nevertheless, none of the hypothesis put forward up to-date, could not confirm a definite cause for CKDu^18^. However, in this study it was able to confirm an existence of a clear correlation between CKDu and the toxic synergic effect of Cd, F and hardness providing a convinced diagnosis for the CKDu confirming our proposed hypothesis in our previous studies^9^,^13^.

## Methods

One hundred and twenty (120) ICR (white) female mice, 3–4 months of age, were used for this study at the start of the treatment schedule. After acclimatization to laboratory animal house conditions for 10 days, the mice were allocated randomly into twelve groups (ten mice/group). Twelve cages were used to house for them with adequate space. All subjects were stock colony at Veterinary Research Institute (VRI) and maintained on a 12:12 h light: dark schedule. Food was provided ad libitum (Commercial broiler starter). Water was also available ad libitum. In this study, animals were divided into twelve groups where in each group 10 ICR mice were treated for twenty eight weeks with the set of conditions shown in Table S1. All experimental protocols were approved by the ethics committee of Postgraduate Institute of Science of University of Peradeniya Sri Lanka and all experiments were performed in accordance with relevant guidelines and regulations stipulated by the ethic committee.

According to WHO report, recommended F, Cd, Pb and As concentrations in drinking water are 1.50, 0.003, 0.010, 0.010 mg/L respectively^19^. Similarly, the secondary WHO standards for Al and hardness in drinking water are (0.200 mg/L)^20^ and (CaCO_3_ > 180 mg/L) respectively. Based on WHO classifications given above, the treated groups were categorized into standard (on par with WHO recommended levels), extreme (above the WHO recommended levels) or control (Table S2). Accordingly, test groups can be divided into three categories; (a) mice treated with the hard water containing other elements in par with WHO standards (groups 2, 4, 6, 12), (b) mice treated with the hard water containing other elements above the WHO standards (groups 3, 8, 9, 10) and (c) mice treated without hard drinking water with elements F, Cd and As; WHO maximum and above (groups 5,7,11) and Group 1 is selected as the control group. During the study period animals were examined routinely for visible clinical signs (reduced appetite, emaciation etc.) including behavioural changes (water intake, feed intake, cannibalism, etc.). The consumption rates of test solutions by mice were monitored during the test period. Initial and final body weights of each group of mice were recorded at the beginning and at the termination of the experiment.

### Treatment solutions

For the preparation of Cd, As, Pb, Al concentrations, 1.00 g/L Cd, As, Pb, Al standards in nitric acids (Fluka, Sigma-Aldrich Chemicals) with serial dilutions were used. For the F concentrations; 1000 mg/L NaF (standard solution of Dionex, Thermo scientific) with serial dilution was used.

### Samples collection and gross examinations

At the termination of the experiment, all surviving animals were euthanized with 5% Ketarmine hydrochloride by injecting intra-muscular dose (150 mg/kg body weight) (‘Ketarmil’, Troy Laborateries pvt ltd, NSW 2164, Australia). Carcasses were examined for external changes such as emaciation and dull coat etc. During the examination of internal organs, the kidneys were examined for gross changes such as size variations, shape, appearance, necrotic or degenerated areas etc. These organs were collected from all the carcasses and immersed in 10% buffered formal saline for histological processing.

### Histopathological evaluations

Fixed tissue samples were embedded in paraffin and sectioned at 4 μm. Sections of kidney and liver tissues were stained with hematoxylin and eosin (H&E). Masson’s trichrome stain was also used to confirm the specific lesions. All sections underwent the same initial steps used in preparing the paraffin-embedded tissue for processing, section de-paraffination, rehydration. Observations of stained sections were made in a 250 × 250 μm area of the particular region of interest under light microscope.

## Additional Information

**How to cite this article**: Wasana, H. M. S. *et al*. WHO water quality standards Vs Synergic effect(s) of fluoride, heavy metals and hardness in drinking water on kidney tissues. *Sci. Rep.* **7**, 42516; doi: 10.1038/srep42516 (2017).

**Publisher's note:** Springer Nature remains neutral with regard to jurisdictional claims in published maps and institutional affiliations.

## Supplementary Material

Supplementary Information

## Figures and Tables

**Table 1:** Average severity % of the treatment groups and control group for considered lesion for CKD.

Lesion type	Severity level	Groups											
		G-1	G-2	G-3	G-4	G-5	G-6	G-7	G-8	G-9	G-10	G-11	G-12
**Interstitium**													
1) Interstitial fibrosis (ci)	ci0	100.0	0.0	0.0	22.2	0.0	0.0	11.1	0.0	0.0	90.0	12.5	33.3
	ci1	0.0	22.2	0.0	77.8	80.0	75.0	89.9	100.0	100.0	10.0	87.5	66.7
	ci2	0.0	77.8	25.0	0.0	20.0	25.0	0.0	0.0	0.0	0.0	0.0	0.0
	ci3	0.0	0.0	75.0	0.0	0.0	0.0	0.0	0.0	0.0	0.0	0.0	0.0
2) Mononuclear cell interstitial infiltration (i)	i0	80.0	0.0	0.0	0.0	0.0	20.0	0.0	0.0	20.0	33.3	0.0	0.0
	i1	20.0	55.6	33.3	89.9	37.5	80.0	77.8	87.5	80.0	66.7	37.5	66.7
	i2	0.0	44.4	66.7	11.1	62.5	0.0	22.2	12.5	0.0	0.0	62.5	33.3
**Tubules**													
3) Tubular atrophy (ct)	ct0	100.0	0.0	0.0	0.0	0.0	0.0	0.0	0.0	12.5	20.0	0.0	0.0
	ct1	0.0	0.0	0.0	22.2	20.0	25.0	66.7	37.5	87.5	80.0	0.0	55.6
	ct2	0.0	62.5	25.0	77.8	80.0	75.0	33.3	62.5	0.0	0.0	89.9	44.4
	ct3	0.0	37.5	75.0	0.0	0.0	0.0	0.0	0.0	0.0	0.0	11.1	0.0
**Glomeruli**													
4) Global glomerular sclerosis score (%)	0	100.0	100.0	0.0	100.0	100.0	100.0	100.0	100.0	100.0	100.0	100.0	100.0
	1–40	0.0	0.0	37.5	0.0	0.0	0.0	0.0	0.0	0.0	0.0	0.0	0.0
	41–65	0.0	0.0	62.5	0.0	0.0	0.0	0.0	0.0	0.0	0.0	0.0	0.0
5) Focal glomerular sclerosis score (%)	0	100.0	77.8	0.0	100.0	100.0	100.0	100.0	100.0	100.0	100.0	100.0	100.0
	1–20	0.0	22.2	37.5	0.0	0.0	0.0	0.0	0.0	0.0	0.0	0.0	0.0
	21–50	0.0	0.0	25.0	0.0	0.0	0.0	0.0	0.0	0.0	0.0	0.0	0.0
	>50	0.0	0.0	37.5	0.0	0.0	0.0	0.0	0.0	0.0	0.0	0.0	0.0
Sample size at the end		10	9	8	9	8	8	9	8	8	10	8	9
