# Supplementary material for: WHO water quality standards Vs Synergic effect(s) of fluoride, heavy metals and hardness in drinking water on kidney tissues
Source: Sci Rep. 2017 Feb 14;7:42516. doi: 10.1038/srep42516 (PMC5307334; doi:10.1038/srep42516)
Supplement: Supplementary Information [file srep42516-s1.doc]

**Supporting Information**

WHO water quality standards Vs Synergic effect(s) of fluoride, heavy metals and hardness in drinking water on kidney tissues

Hewa M.S.Wasana^1^, Gamage D.R.K. Perera^2^, **Panduka De S. Gunawardena^3^, Palika S. Fernando^4^,**Jayasundera Bandara^1^*

**Materials and Methods**

For this work, ethical clearance was obtained from the ethical clearance committee in Postgraduate Institute of Science, University of Peradeniya, Sri Lanka.In this study, animals were divided into twelve groups where in each group 10 ICR mice were treated for twenty six weeks with the following set of conditions (Table S1)

Table S1: Experimental details of test solutions for the mice.

| Group | Combinations/ Elements | Concentrations (mg/L) |
| --- | --- | --- |
|  |
| 1 | Control (Kandy tap water) | CaCO_3_ + MgCO_3 =_55.0, F=0.06, Al=0.160, Cd=0.00008 |
| 2 | Cd ,F, Hardness | F= 1.5, Cd=0.003, CaCO_3_ = 200.0, MgCO_3_= 185.0 |
| 3 | Cd, F, Hardness | F= 3.0, Cd=0.006, CaCO_3_ = 400.0, MgCO_3_= 370.0 |
| 4 | Cd, Hardness | Cd=0.003, CaCO_3_ = 200.0, MgCO_3_= 185.0 |
| 5 | Cd, F | F= 1.5, Cd=0.003 |
| 6 | F, Hardness | F= 1.5, CaCO_3_ = 200.0, MgCO_3_= 185.0 |
| 7 | Cd | Cd= 0.006 |
| 8 | As, F, Hardness | As=0.015, F= 3.0,CaCO_3_ = 200.0, MgCO_3_= 185.0 |
| 9 | Pb, F, Hardness | Pb=0.015, F= 3.0, CaCO_3_ = 200.0, MgCO_3_= 185.0 |
| 10 | Al, F, Hardness | Al=0.200, F= 3.0, CaCO_3_ = 200.0, MgCO_3_= 185.0 |
| 11 | As | As=0.015 |
| 12 | Al, Cd, As, Pb, F, Hardness | Al=0.200, Cd=0.003, As=0.010, Pb=0.010, F= 1.5, CaCO_3_ = 200.0, MgCO_3_=185.0 |

According to WHO recommended F, Cd, Pb and As concentrations in drinking water and the secondary WHO standards for Al and hardness in drinking water, the treated groups were categorised into standard, extreme or control and the details are given in Table S2.

Table S2: Treatment solutions for mice, compared to WHO standards.

| Group | Combinations/ Elements | Cd | F | Hardness | As | Pb | Al |
| --- | --- | --- | --- | --- | --- | --- | --- |
|  |  |  |  |  |  |
| 1 | Control (Kandy tap water) | C | C | C | - | - | C |
| 2 | Cd ,F, Hardness | S | S | E | - | - | - |
| 3 | Cd, F, Hardness | E | E | E | - | - | - |
| 4 | Cd, Hardness | S | - | E | - | - | - |
| 5 | Cd, F | S | S | - | - | - | - |
| 6 | F, Hardness | - | S | E | - | - | - |
| 7 | Cd | E | - | - | - | - | - |
| 8 | As, F, Hardness | - | E | E | E | - | - |
| 9 | Pb, F, Hardness | - | E | E | - | E | - |
| 10 | Al, F, Hardness | - | E | E | - | - | S |
| 11 | As | - | - | - | E | - | - |
| 12 | Al, Cd, As, Pb, F, Hardness | S | S | E | S | S | S |

S-Standard; WHO maximum standard, (F=1.5 mg/l, Cd=3 µg/l, As=10 µg/l, Pb=10 µg/l, secondary standard Al=200 µg/l)

E-Extreme; Exceeding WHO maximum standard up to double concentrated for particular element.

C- Control; well below the WHO standard

Table S3: Average water intake, body weight (loss/gain), death counts and duration for deaths; of the mice in test groups and control group.

| Group | Average water intake | Avg body weight | Early deaths | Duration for deaths (Days) |
| --- | --- | --- | --- | --- |
|  | (mL/week/mice) | (Loss/Gain) |  |  |
|  |  | per mice(g) |  |  |
| 1 | 32 | 9 | 0 | 0 |
| 2 | 30 | -3 | 1 | 99 |
| 3 | 30 | -7 | 2 | 63,82 |
| 4 | 31 | -4 | 1 | 98 |
| 5 | 29 | -5 | 2 | 92,125 |
| 6 | 32 | -3 | 2 | 108,130 |
| 7 | 30 | -3 | 1 | 111 |
| 8 | 31 | -5 | 2 | 96,103 |
| 9 | 30 | -3 | 2 | 105,134 |
| 10 | 30 | -1 | 0 | 0 |
| 11 | 32 | -5 | 2 | 90,105 |
| 12 | 31 | -3 | 1 | 140 |

| **Post-mortem finding** | **G-1** | | **G-2** | | **G-3** | | **G-4** | | **G-5** | | **G-6** | | **G-7** | | **G-8** | | **G-9** | | **G-10** | | **G-11** | | **G-12** | |
| --- | --- | --- | --- | --- | --- | --- | --- | --- | --- | --- | --- | --- | --- | --- | --- | --- | --- | --- | --- | --- | --- | --- | --- | --- |
|  | **D** | **H** | **D** | **E** | **D** | **E** | **D** | **H** | **D** | **E** | **D** | **H** | **D** | **E** | **D** | **E** | **D** | **E** | **D** | **E** | **D** | **E** | **D** | **E** |
| 1. Necrotic foci on kidney/s | 0/0 | 0/0 | 1/1 | 4/9 | 2/2 | 6/8 | 1/1 | 2/9 | 2/2 | 3/8 | 1/2 | 3/8 | 1/1 | 3/9 | 1/2 | 2/8 | 1/2 | 2/8 | 0/0 | 0/10 | 1/2 | 2/8 | 0/1 | 0/9 |
| 2) Hepatomegaly | 0/0 | 0/0 | 1/1 | 5/9 | 2/2 | 6/8 | 1/1 | 3/9 | 2/2 | 5/8 | 1/2 | 4/8 | 1/1 | 4/9 | 2/2 | 5/8 | 1/2 | 4/8 | 0/0 | 3/10 | 1/2 | 5/8 | 1/1 | 5/9 |
| 3) Necrotic foci on liver lobes | 0/0 | 0/0 | 1/1 | 3/9 | 2/2 | 5/8 | 1/1 | 2/9 | 2/2 | 3/8 | 0/2 | 0/2 | 1/1 | 2/9 | 2/2 | 1/8 | 1/2 | 2/8 | 0/0 | 0/10 | 1/2 | 1/8 | 0/1 | 0/9 |
| 4) Enlarged lymph nodes | 0/0 | 0/0 | 1/1 | 3/9 | 2/2 | 6/8 | 1/1 | 3/9 | 2/2 | 4/8 | 1/2 | 2/8 | 1/1 | 4/9 | 2/2 | 5/8 | 2/2 | 3/8 | 0/0 | 2/10 | 2/2 | 5/8 | 1/1 | 5/9 |
| 5) Splenomegaly | 0/0 | 1/0 | 1/1 | 6/9 | 2/2 | 7/8 | 1/1 | 4/9 | 2/2 | 4/8 | 2/2 | 3/8 | 1/1 | 4/9 | 2/2 | 5/8 | 2/2 | 4/8 | 0/0 | 4/10 | 2/2 | 5/8 | 1/1 | 4/9 |
| 6) Alopecia (a) submandibular | 0/0 | 0/0 | 0/1 | 0/9 | 0/2 | 0/8 | 0/1 | 0/9 | 0/2 | 0/8 | 0/2 | 0/8 | 0/1 | 0/9 | 1/2 | 4/8 | 0/2 | 0/8 | 0/0 | 0/10 | 2/2 | 4/8 | 0/1 | 0/9 |
| 6) Alopecia (b) rostral | 0/0 | 0/0 | 0/1 | 0/9 | 0/2 | 0/8 | 0/1 | 0/9 | 0/2 | 0/8 | 0/2 | 0/8 | 0/1 | 0/9 | 0/2 | 2/8 | 0/2 | 0/8 | 0/0 | 0/10 | 0/2 | 3/8 | 0/1 | 0/9 |

**Table S4: Gross evaluations/post-mortem evaluations of mice in test groups and the control group at the end of the experiment.**

**D**-natural deaths of mice.

**E**-Euthanized mice at the end of the experiment.

**Numerator**- number of positive animals in considered group for particular lesion.

**Denominator**- number of total deaths/euthanasia at the end of the study.

**Working classification**

**Criteria for histopathological analysis**

**Interstitium**

**1) Quantitative criteria for interstitial fibrosis (‘‘ci’’)**

ci0- Interstitial fibrosis in up to 5% of cortical area

ci1 -Mild-interstitial fibrosis in 6–25% of cortical area

ci2 -Moderate-interstitial fibrosis in 26–50% of cortical

ci3- Severe-interstitial fibrosis in >50% of cortical area

**2) Quantitative criteria for mononuclear cell interstitial inflammation ("i")**

i0- No or trivial interstitial inflammation <10% of unscarred parenchyma

i1- 10–25% of parenchyma inflamed

i2- 26–50% of parenchyma inflamed

i3- More than 50% of parenchyma inflamed

**Tubules**

**3) Quantitative criteria for tubular atrophy ("ct")**

ct0- No tubular atrophy

ct1 - tubular atrophy in up to 25% of the area of cortical tubules

ct2- tubular atrophy involving 26-50% of the area of cortical tubules

ct3 - tubular atrophy in >50% of the area of cortical tubules

**4) Apoptosis (a)**

a0 - apoptosis < 5 % in the cells of cortical area

a1 - apoptosis 6- 25 % in the cells of cortical area

a2 - apoptosis 26-50 % in the cells of cortical area

a3 - apoptosis > 50 % in the cells in cortical area

**5) Cell degeneration (d)**

d0 - cell degradation < 5 % in the cells of cortical area

d1 - cell degradation 6- 25 % in the cells of cortical area

d2 - cell degradation 26-50 % in the cells of cortical area

d3 - cell degradation > 50 % in the cells in cortical area

**6) Cell necrosis (n)**

n0 - cell necrosis < 5 % in the cells of cortical area

n1 - cell necrosis 6- 25 % in the cells of cortical area

n2 - cell necrosis 26-50 % in the cells of cortical area

n3 - cell necrosis > 50 % in the cells in cortical area

**Glomerular lesions**

**7) Global glomerulosclerosis score** = (sclerotic glomeruli/total glomeruli) x100%

**8) Focal segmental glomerularsclerosis count** = (Segmental sclerotic glomeruli/total glomeruli) x100%

**Vasculature**

**9) Hyperemia (hy)**

hy0 - Hyperemia<5 % in blood vessels in parenchyma

hy1 - Hyperemia 6-25 % in blood vessels in parenchyma

hy2 - Hyperemia 26-50 % in blood vessels in parenchyma

hy3 - Hyperemia > 50 % in blood vessels in parenchyma

**10) Hemorrhage (he)**

he0 - hemorrhage <5 % in blood vessels in parenchyma

he1 - hemorrhage 6-25 % in parenchyma

he2 - hemorrhage 26-50 % in parenchyma

he3 - hemorrhage > 50 % in parenchyma

| **Lesion** |  | | | | | | | | | | | | |
| --- | --- | --- | --- | --- | --- | --- | --- | --- | --- | --- | --- | --- | --- |
|  | **Severity** | G-1 | G-2 | G-3 | G-4 | G-5 | G-6 | G-7 | G-8 | G-9 | G-10 | G-11 | G-12 |
| 6) Apoptosis (a) | a0 | 5.0 | 0.0 | 0.0 | 0.0 | 0.0 | 0.0 | 0.0 | 0.0 | 0.0 | 0.0 | 0.0 | 0.0 |
|  | a1 | 80.0 | 22.2 | 12.5 | 22.2 | 37.5 | 25.0 | 22.2 | 25.0 | 12.5 | 80.0 | 50.0 | 100.0 |
|  | a2 | 15.0 | 77.8 | 87.5 | 77.8 | 62.5 | 75.0 | 77.8 | 75.0 | 87.5 | 20.0 | 50.0 | 0.0 |
| 7) Cell degeneration (d) | d0 | 0.0 | 0.0 | 0.0 | 0.0 | 0.0 | 0.0 | 0.0 | 0.0 | 0.0 | 0.0 | 0.0 | 0.0 |
|  | d1 | 90.0 | 33.3 | 12.5 | 22.2 | 37.5 | 25.0 | 44.4 | 25.0 | 12.5 | 80.0 | 12.5 | 44.4 |
|  | d2 | 10.0 | 66.7 | 87.5 | 77.8 | 62.5 | 75.0 | 55.6 | 75.0 | 87.5 | 20.0 | 87.5 | 55.6 |
| 8) Cell necrosis (n) | n0 | 90.0 | 0.0 | 0.0 | 0.0 | 0.0 | 0.0 | 0.0 | 0.0 | 0.0 | 60.0 | 0.0 | 0.0 |
|  | n1 | 10.0 | 44.4 | 0.0 | 55.6 | 25.0 | 75.0 | 66.7 | 37.5 | 100.0 | 40.0 | 12.5 | 77.8 |
|  | n2 | 0.0 | 55.6 | 62.5 | 44.4 | 75.0 | 25.0 | 33.3 | 62.5 | 0.0 | 0.0 | 87.5 | 22.2 |
|  | n3 | 0.0 | 0.0 | 37.5 | 0.0 | 0.0 | 0.0 | 0.0 | 0.0 | 0.0 | 0.0 | 0.0 | 0.0 |
| 9) Hyperemia (hy) | cn0 | 80.0 | 0.0 | 0.0 | 0.0 | 0.0 | 0.0 | 0.0 | 0.0 | 0.0 | 0.0 | 0.0 | 0.0 |
|  | cn1 | 20.0 | 0.0 | 0.0 | 0.0 | 0.0 | 50.0 | 44.4 | 0.0 | 0.0 | 0.0 | 0.0 | 0.0 |
|  | cn2 | 0.0 | 100.0 | 0.0 | 100.0 | 100.0 | 50.0 | 55.6 | 37.5 | 50.0 | 80.0 | 0.0 | 33.3 |
|  | cn3 | 0.0 | 0.0 | 100.0 | 0.0 | 0.0 | 0.0 | 0.0 | 62.5 | 50.0 | 20.0 | 100.0 | 66.7 |
| 10) Hemorrhage (he) | he0 | 10.0 | 0.0 | 0.0 | 0.0 | 0.0 | 0.0 | 0.0 | 0.0 | 0.0 | 0.0 | 0.0 | 0.0 |
|  | he1 | 90.0 | 0.0 | 0.0 | 33.3 | 50.0 | 75.0 | 33.3 | 0.0 | 0.0 | 70.0 | 0.0 | 33.3 |
|  | he2 | 0.0 | 44.4 | 37.5 | 66.7 | 50.0 | 25.0 | 66.7 | 62.5 | 75.0 | 30.0 | 50.0 | 66.7 |
|  | he3 | 0.0 | 55.6 | 62.5 | 0.0 | 0.0 | 0.0 | 0.0 | 37.5 | 25.0 | 0.0 | 50.0 | 0.0 |

**Table S5: Analysed extraLesion’s %, of positive animals in test groups, from the survivals at the end of the study.**

**Table S6: Severity order of the groups for considered lesions.**

| Lesion | Severity group order |
| --- | --- |
| 1) Interstitial fibrosis (multifocal) (ci) | G3>G2>G6>G5>G8,G9>G11>G7>G4>G12>G10>G1 |
| 2) Mononuclear cell interstitial infiltration(i) | G3>G11>G2>G5>G12>G7>G8>G4>G9,G6>G10>G1 |
| 3) Tubular atrophy(ct) | G3>G2>G11>G5>G6>G4>G8>G12>G7>G9>G10>G1 |
| 4) Apoptosis (a) | G3>G2,G4,G7>G5>G9>G6,G8>G11>G12>G10>G1 |
| 5) Cell degeneration (d) | G3,G11>G5>G2,G4>G7>G8>G12>G9>G6>G10>G1 |
| 6) Cell necrosis (n) | G3>G11>G5>G2>G8>G4>G7>G6>G12>G9>G10>G1 |
| 7) Global glomerular sclerosis score% | G3 only |
| 8) Focal segmental glomerular sclerosis% | G3>G2 |
| 9) Hyperaemia (hy) | G3,G11>G12>G8>G9>G2,G5,G4>G6>G7>G10>G1 |
| 10) Haemorrhages (he) | G3>G11>G8>G2>G9>G7>G12>G5>G4>G10>G6>G1 |

**Plot of Figure 2**

The Figure 2 was plotted by using "OriginPro 9" software with  contour- color fill 2D plot. For the plot of Figure 2, treatment groups 1 to 12 were taken as X axis and for Y axis, lesions types 1,2,3,5 and 5 were selected. (1. Interstitial fibrosis (ci) ,2. Mono-nuclear cell interstitial infiltration (i), 3.Tubular atrophy (ct), 4. Focal glomerular sclerosis and 5. Global glomerular sclerosis. The Data table (S7), which used to draw the figure 2 was given in excel sheet. (S7)

**References:**

1. Organization, W.H.*, Guidelines for Drinking-water Quality (3rd edn. incorporating the first addendum). Volume 1. Recommendation*s. World Health Organization, Geneva, 2006: p. 1-5.


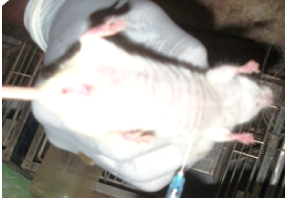

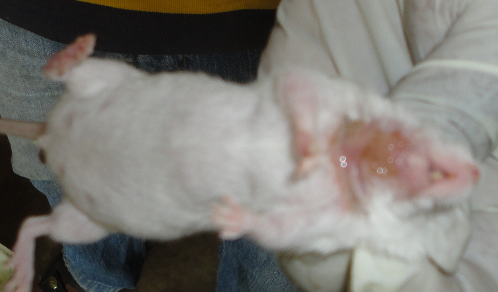

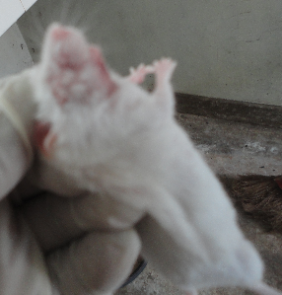

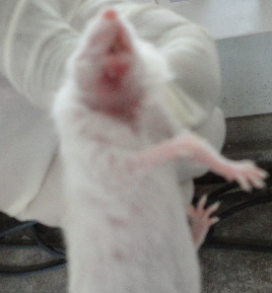


1. (B) (C) (D)

Figure S1: Pruritic and focal (submandibular,rostral) alopecic areas (arrows) of affected groups; A and B- G11, C- G8. D- Control group (G1) with intact skin


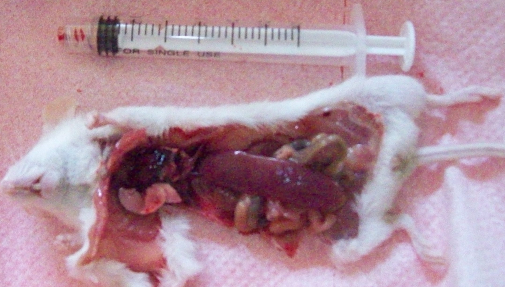

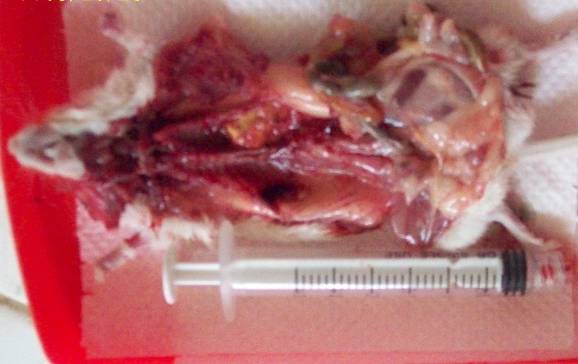


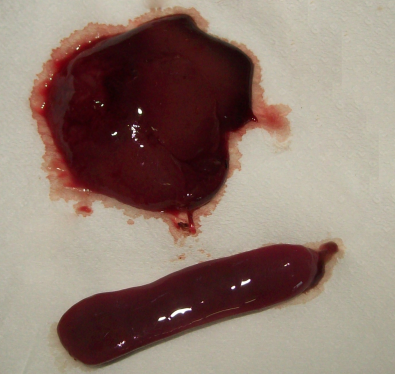

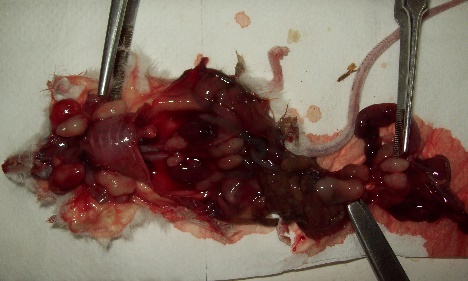
 (A) (B)


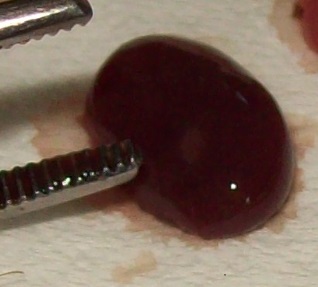


1. (D) (E)

Figure S2: Gross post-mortem findings in G3, (A)-necrotic foci (arrow) on kidney, (B)- Splenomegaly (arrow), (C)- Enlarged lymph nodes (sub mandibular, axillary, mesenteric), (D) heptaomegaly and splenmegaly and (E) appearance of lymph nodes (arrows) in control group (G1).

**
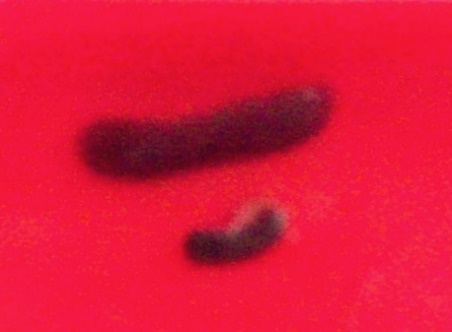
**

G3

G1

Figure S3: Comparison of spleens in G3 with G1 (control)
